# Towards prospective identification of optimal scan parameters for CT-guided interventions: a phantom study

**DOI:** 10.1038/s41598-026-65962-y

**Published:** 2026-08-07

**Authors:** Constantin Schareck, Neele Blumhoff, Malte M. Sieren, Roman Kloeckner, Franz Wegner

**Affiliations:** 1 Institute for Interventional Radiology, University of Lübeck, Ratzeburger Allee 160, 23538 Lübeck, Germany; 2 Institute of Radiology and Nuclear Medicine, University of Lübeck, Ratzeburger Allee 160, 23538 Lübeck, Germany; 3 Fraunhofer Research Institution for Individualized Medical Technology and Engineering IMTE, Mönkhofer Weg 239a, 23562 Lübeck, Germany

**Keywords:** Interventional radiology, Radiation protection, Medical imaging, Contrast to noise ratio, Size specific dose estimate, Engineering, Health care, Medical research, Physics

## Abstract

CT-guided interventions require balance between radiation dose and image quality. This study evaluates the impact of tube voltage and tube load on the contrast-to-noise ratio (CNR) across different patient sizes and target tissues. A modular multi-energy phantom with effective diameters of 20 cm and 35 cm was used. Bone, iodine-blood mixture, and pure blood targets were scanned axially at matched dose levels. Images were equally reconstructed. For analysis, CNR was the primary metric. Tube voltages of 80, 100, 120 and 140 kVp were applied with corresponding mAs adjustments to achieve dose levels between 1 and 10 mGy SSDE (Size Specific Dose Estimate). In the small phantom, low kVp settings yielded the highest CNR for high-contrast targets like iodine. For low-contrast imaging, higher kVp settings were more beneficial. In the large phantom, higher kVp consistently improved CNR across all materials. E.g. raising kVp from 80 to 120 raised the CNR from 6.5 to 8.6 for the iodine-blood setup. In conclusion, for optimal image quality in CT-guided interventions, scan protocols must adapt tube voltage and tube load dynamically to the target tissue and patient size. Finding the sweet spot between contrast loss and noise reduction for increasing tube voltages is key for intervention planning.

## Introduction

CT guided interventions are widely established diagnostic and therapeutic procedures. To accurately perform an intervention, the tissue of interest (TOI, also referred to as the target) must be clearly identified. This requires balancing radiation dose and image quality to ensure optimal visualization while minimizing radiation-related risks. The challenge is particularly pronounced when the TOI exhibits low contrast relative to surrounding tissues. In comparison, higher contrast differences allow for lower image quality and reduced radiation exposure. This ‘contrast classification’ can guide the interventionalist in selecting appropriate CT examination parameters prior to the procedure [1].

Since CT-Interventions do not have to meet the same standards applied to diagnostic imaging, lower dose levels than the prior diagnostic planning CT can be used [2, 3]. The current–time product of the X-ray tube is linear and the tube voltage contributes exponentially to the CT-dose [3, 4]. A change in CT dose due to a change in either kVp or mAs can therefore be counteracted by changing both parameters simultaneously. In principle, dose changes resulting from one parameter can be compensated by adjusting the other. Consequently, low-kVp protocols are widely recommended to enhance image contrast and reduce patient dose [2]. However, this one-size-fits-all approach often fails in clinical practice. While lower kVp values increase contrast, particularly for iodine-enhanced targets, they also substantially increase image noise, especially in larger patients. This can negate the expected contrast benefit and result in suboptimal visualization of the target tissue. Moreover, low-kVp beams may lack sufficient penetration in larger body regions, further degrading image quality [5–7]. The resulting suboptimal settings often result in repeated scans, unnecessarily increasing both radiation dose and intervention time.

The aim of this study was to identify optimal scan parameters under varying conditions. Specifically, how tube voltage affects CNR, and whether scan parameters should be adapted to patient size and tissue type instead of using fixed low-dose settings.

## Material and methods

### Phantom Set up

**Fig. 1:**
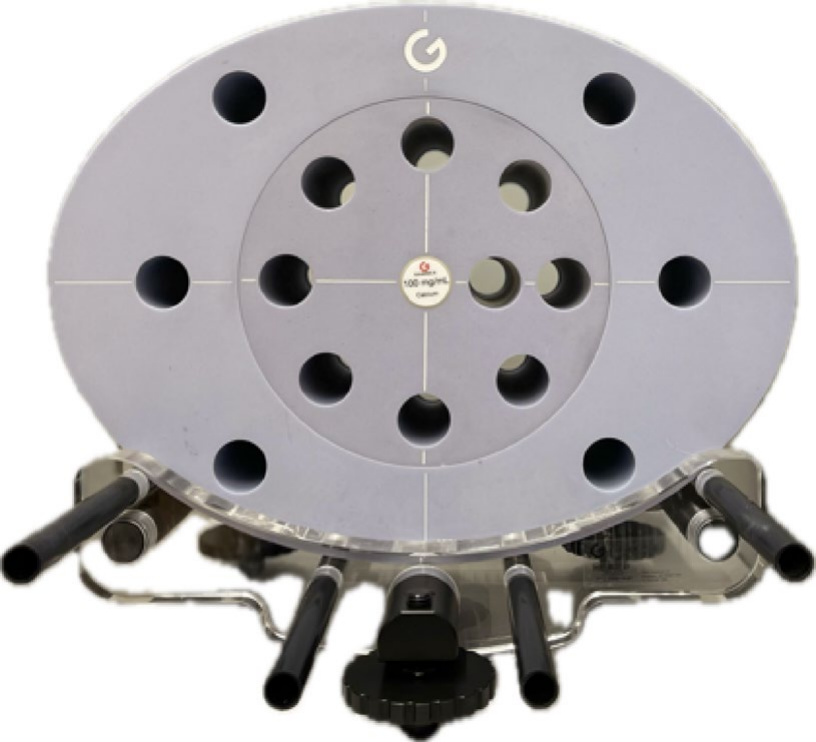
The Sun Nuclear Multi-Energy Phantom features insertions holes designed to accommodate probes of varying densities that mimic different tissue types. Notably, the phantom includes a detachable middle section, allowing scans to be performed with an effective diameter of either 20 cm or 35 cm. Rods composed of different materials, each measuring 16.5 cm in length, were consistently positioned at the center.

**Fig. 2:**
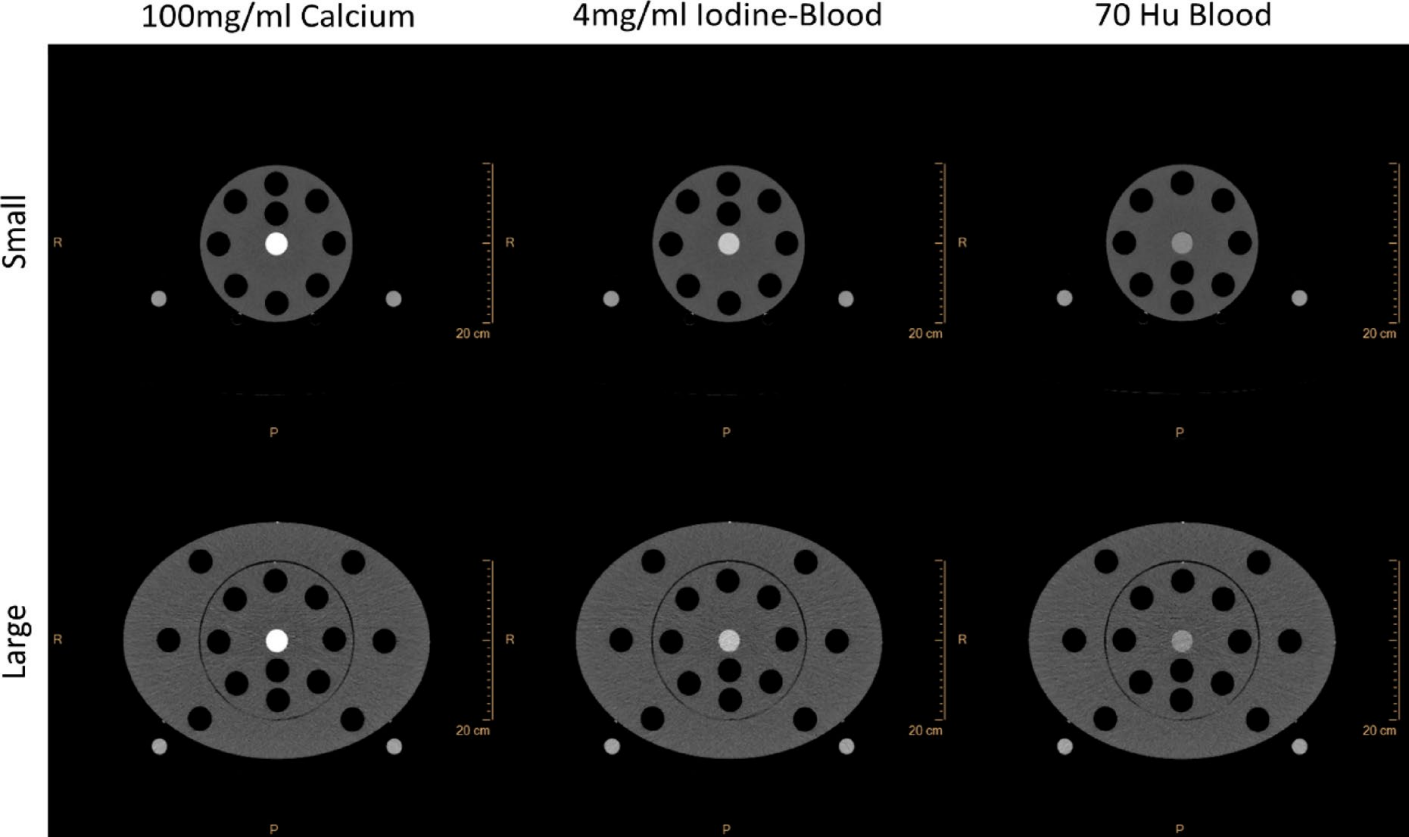
Exemplary CT scans of 100 mg/ml calcium, 4 mg/ml Iodine-Blood and Blood 70 HU (from left to right) scanned with 20 cm phantom (first row) and with the 35 cm phantom (second row). The window leveling was W:350/C:60.

CT scans (CT7500, Philips Healthcare, The Netherlands) of a multi-energy phantom (Sun Nuclear Melbourne, Florida) were performed (see Fig. 1). The influence of high and low contrast structures on CT-dose for sufficient image quality in CT-guided interventions was investigated. The phantom can be equipped with rods of different densities. We used rods with HU-values comparable to 4 mg/ml Iodine-blood (~ 120–220 HU), pure blood (normed to 70 HU) and 100 mg calcium/ml (~ 300–400 HU). The material choice was made to address the most common abdominal biopsy procedures, such as bone and parenchymal organ biopsies (e.g. liver and kidney) and a possible necessary administration of contrast agents. The rods were placed in the middle of the phantom to reach an equal distribution of beam hardening effects. All left over wholes were not filled with rods of different material to avoid measurement bias and keep the comparison base equal between scans of the different rods. Since the phantom is built modular by means of its effective diameter, i.e. the geometric mean of the phantoms’ dimensions frontal and lateral, scans were performed twice for each rod. Once for an effective diameter of 20 cm (representing a pediatric or slim adult patient) and second for an effective diameter of 35 cm (representing a standard adult patient) (see Fig. 2).

### Scan parameters

The CT scans were performed with tube voltages of 80 kVp, 100 kVp, 120 kVp and 140 kVp. The amount of radiation (measured in mAs) was adjusted based on the selected tube voltage (kVp), so that the starting dose for each scan was 1 mGy. KVp and mAs were used for this study as these are the most prominent scan-parameters adjusted by the interventionalist in the case of insufficient image quality. With each new scan, the dose was increased by 1 mGy, up to a maximum of 10 mGy. If a 1 mGy dose wasn’t possible for a particular voltage setting, we used the lowest dose that could be achieved. Because the standard dose measurement (CTDIvol) for body scans is based on measurements of a 32 cm cylindric phantom, we corrected for the actual size of the models used in our study by calculating a more accurate value called the Size-Specific Dose Estimate (SSDE) as shown in Table 1 [8]. Adjusting the tube voltage towards higher kVp comes with the necessity to counteract the accompanied dose increasement by lowering the mAs to keep the CTDI_Vol/SSDE similar. Otherwise the patient would be unintentionally and unjustified exposed to more radiation. It should always be in mind that the higher the kVp is set the higher the minimum possible CTDI_Vol/SSDE becomes. This leads to the expectation that high kVp can reduce the patient’s exposition, but only if the currently used dose profile is greater than or equal to the minimum dose profile for the next higher voltage. Reconstruction parameters were always set to perform with a slice thickness of 3 mm and a smoothing convolution (B-)filter. A hybrid iterative reconstruction (IR) with levels 0 and 5 (iDose, Philips Healthcare, Netherlands) were both used as reconstruction algorithms to achieve insight towards the increase in CNR by the noise reduction potential of IR for interventional scans.

**Table 1:** Example conversion of the CTDI_Vol_32_ value used for scans of the small phantom into the SSDE value. The conversion factor was defined at 1.78 for effective diameters of 20 cm. It is easy to see that the SSDE nearly doubles. Thus, the importance of using SSDE instead of CTDI_VOL_32_ for small patient scanning becomes clear.

Scan	CTDI_VOL_32_ for small phantom scans [mGy]	SSDE_20_(CTDI_VOL_32_)
1	0.5	0.9
2	1.1	2
3	1.6	2.9
4	2.2	3.9
5	2.7	4.8
6	3.2	5.7
7	3.8	6.8
8	4.3	7.7
9	4.8	8.5
10	5.4	9.6

### Data analysis

**Fig. 3:**
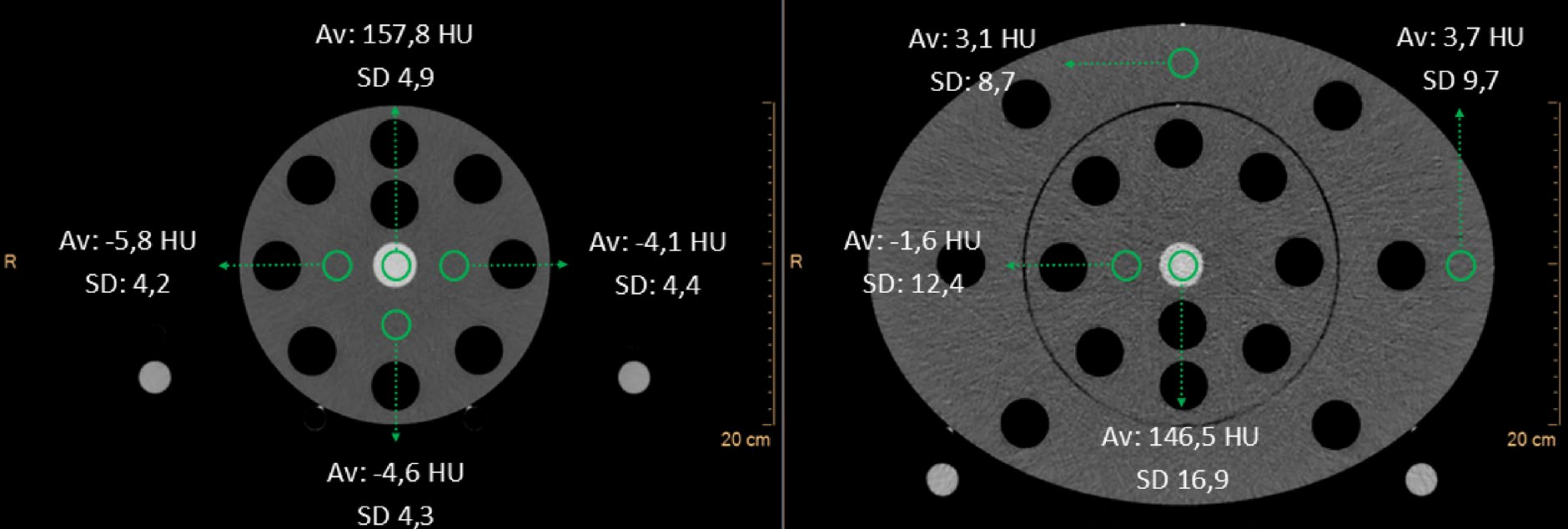
Exemplary representation of the ROI position for the data analysis in the image for the same scan with the iodine-blood mixture rod for the small phantom on the left and the large phantom on the right.

We used the CNR to measure image quality. CNR considers both how much contrast there is in the image and how much visual noise is present, which can vary depending on the tube voltage used during the scan. To measure this, we placed small areas of interest in both the target area and surrounding tissues (see Fig. 3).

Since certain imaging effects, like beam hardening, are influenced by how thick the object being scanned is, we added three more ROIs next to the target and in the phantom’s periphery. To adjust for these effects, we gave more weight (2/3) to the values from the edges of the phantom and less weight (1/3) to the center when calculating the reference signal used for the CNR. This method follows the guidelines set out in the EN IEC 61,223–3-5:2019 + AC:2022 standard, which covers image noise, uniformity, and average CT values.

Finally, we plotted the CNR values against the corresponding SSDE for each voltage setting and phantom size using Matlab (R2024a, MathWorks, Natick, Massachusetts, USA).

## Results

### Small phantom

In the smaller phantom setup with an effective diameter of 20 cm, the influence of tube voltage on CNR was strongly dependent on the type of contrast within the scanned material. For high-contrast targets such as iodine-enriched structures, the application of low tube voltage—particularly 80 kVp—resulted in the highest CNR values across the entire tested dose range (1–10 mGy SSDE). This voltage setting consistently outperformed all others in terms of contrast depiction in iodine-blood mixtures, with CNR values notably elevated even at the lowest dose levels. Similarly, calcium-containing rods demonstrated favorable CNRs at 80 kVp, which remained on par with those obtained at 100 kVp for SSDE levels up to approximately 7 mGy. Beyond this point, 100 kVp scans began to show a modest advantage in calcium imaging, especially at higher dose settings.

**Fig. 4:**
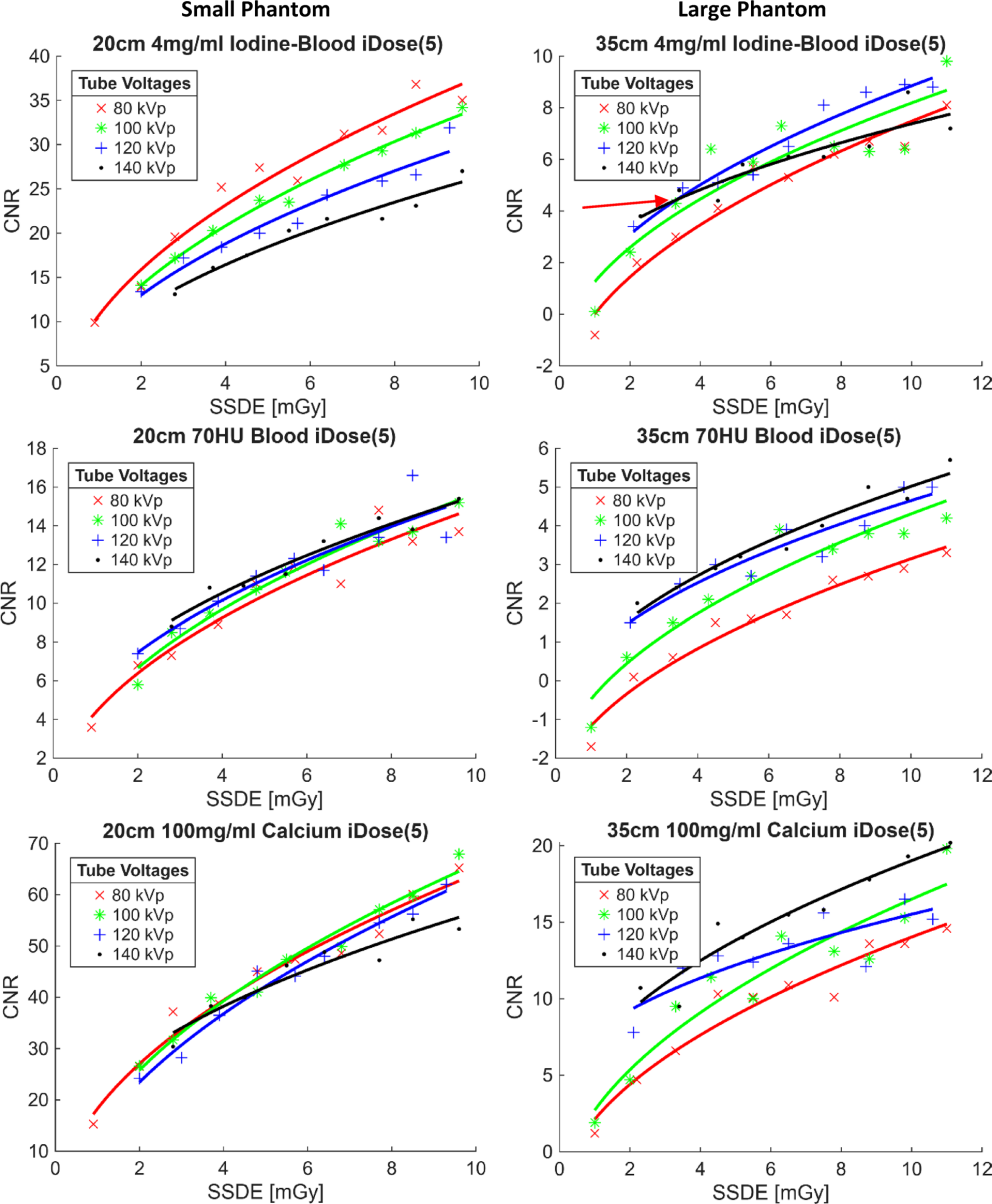
Plots of the CNR course for each used rod once scanned in the small and once in the large phantom. The influence of the change in diameter and therefore of the patient’s constitution becomes clear. The axis limitations were set to fit the maximum occurring CNR for one scan setup. The red arrow indicates dose levels where a change in kVp would lead to a better CNR than simply increasing the tube load. Thus, a recommendation for kVp-settings depending on the TOI and object diameter can be derived.

In contrast, for low-contrast targets such as unenhanced blood-equivalent material, low tube voltage settings proved suboptimal CNR. The CNR for blood-equivalent targets was lowest at 80 kVp throughout the dose range. Instead, higher tube voltages such as 120 kVp and 140 kVp yielded markedly improved results. In particular, 140 kVp scans achieved the highest CNR values for low-contrast structures at SSDEs of 3 mGy and above. Below this threshold, 120 kVp scans were superior, 140 kVp scanning was technically limited to a minimum dose level of 3 mGy in the present setup (see Fig. 4).

### Large phantom

In the large phantom, which was configured with an effective diameter of 35 cm, the relationship between tube voltage, image contrast, and image noise demonstrated a markedly different pattern. Across all contrast types and dose levels, the lowest tube voltage setting (80 kVp) consistently resulted in the lowest CNR values. This was observed even for iodine-enriched samples.

For high-contrast structures such as calcium and iodine-blood mixtures, higher tube voltage settings led to significantly improved image quality. In the case of iodine-enriched targets, the use of 140 kVp produced the highest CNRs at low dose levels (approximately 2 to 4 mGy SSDE). Beyond this range, scans performed at 120 kVp surpassed 140 kVp in terms of CNR. Similar trends were observed for calcium-containing rods, with 140 kVp achieving the highest CNR across nearly the entire SSDE range tested. Notably, at no point did 80 kVp produce competitive results for high-contrast targets in the large phantom.

Low-contrast targets such as unenhanced blood structures showed a similar dependency. Here, both 120 kVp and 140 kVp significantly outperformed the lower settings, with 140 kVp providing the highest CNR values throughout (see Fig. 4).

### Influence of iterative reconstruction

Although not the primary focus, we additionally analyzed the effect of iterative reconstruction on image quality. Using Philips’ iDose algorithm, two levels were compared: iDose0 (filtered back projection) and iDose5 (iterative reconstruction). Across all phantom sizes, voltages, and contrast types, iDose5 yielded ~ 34% higher CNR, mainly through noise reduction. The overall shape of the CNR curves remained unchanged, indicating that reconstruction primarily affects noise rather than tissue contrast.

**Fig. 5:**
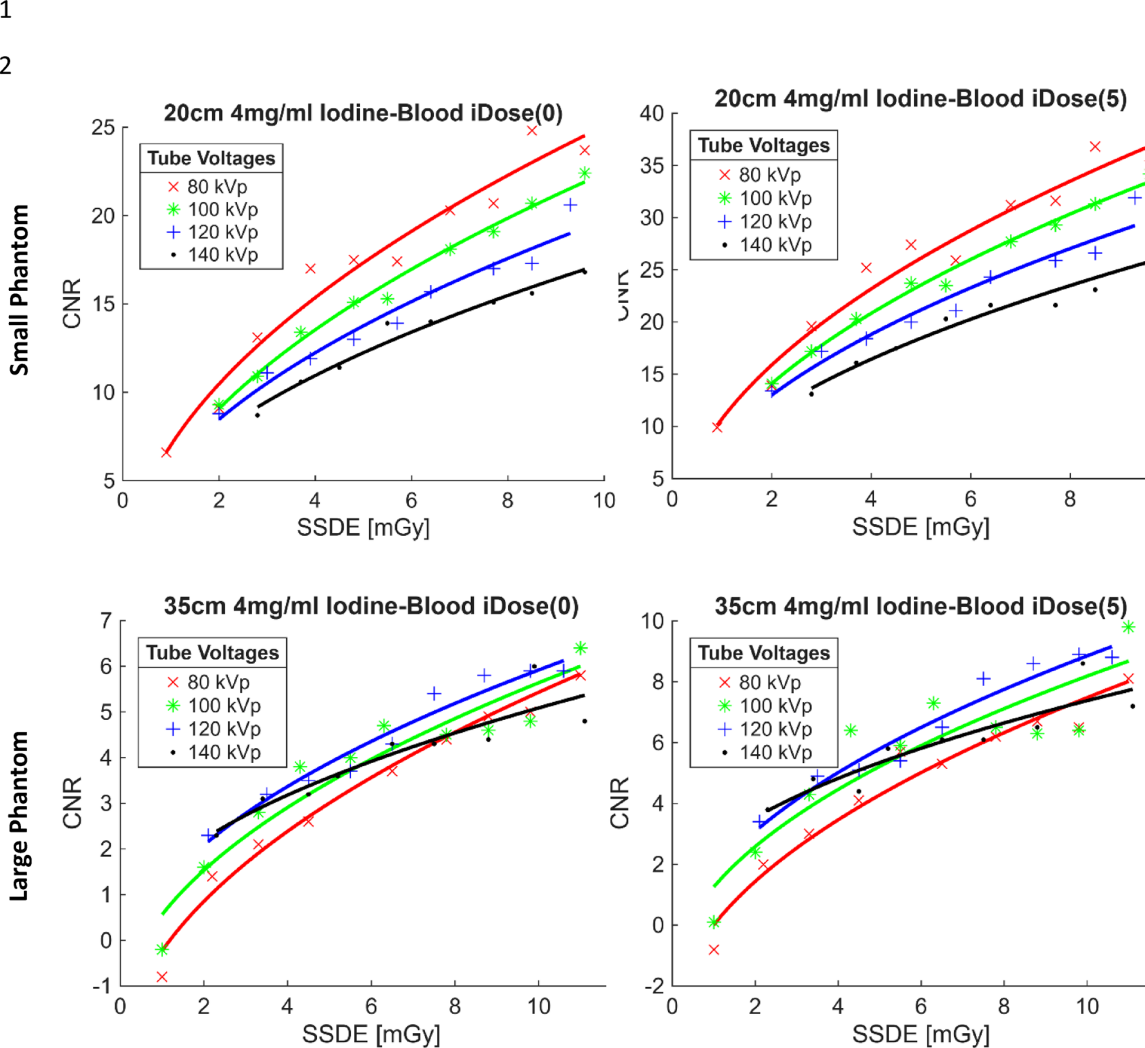
Plots of iodine-blood scans in the small and large phantom with iDose(0) and iDose(5) reconstruction levels. *Note*: The scale was chosen respectively for each plot, such that the difference in CNR for each scan is discernible. As one can see iDose(5) yield increased CNR due to its more powerful denoising probability, while the overall CNR course remains similar. Thus, iDose(5) results are more suitable for the purpose of optimizing CNR for CT-guided interventions due to its noise reduction property while keeping the contrast nearly the same.

Thus, iterative reconstruction enhances image quality but operates largely independently of tube voltage optimization. It elevates overall CNR amplitude without altering the relative performance of different voltage settings. Therefore, results from both modes were excluded from the core analysis but presented separately (see Fig. 5) to illustrate denoising potential while preserving contrast trends.

## Discussion

In this study, we experimentally developed scan-parameter recommendations for various CT intervention settings. While no universal set of parameters exists, systematic trends can be identified. For small phantoms, high contrast imaging achieved the highest CNR at lower kVp settings, whereas low contrast imaging benefitted from 140 kVp. Scans of the large phantom showed increasing CNR with 140 kVp.

The question guiding our study was: *What is the optimal tube voltage for low-dose CT-guided interventions?* Choosing between 80, 100, 120, or 140 kVp depends strongly on patient size and tissue contrast. Low dose scans come with reduced photon intensity, making even a child’s body a strong absorber. Thus, maximizing detected photons is crucial. To investigate body size dependency, we used phantoms of 20 cm and 35 cm effective diameter, representing a 10 year old child (~ 30 kg) and an adult (> 80 kg) [9].

In the smaller phantom, 140 kVp scans yielded the highest CNR for low-contrast targets such as soft tissue. Noise reduction at higher kVp outweighed the expected contrast loss, which is consistent with previous findings by Nagel et al. [3]. Contraire, both image noise and insufficient beam penetration of low kVp settings significantly reduced image quality. For high contrast TOIs like bone or iodine-blood mixtures, lower voltages provided higher CNR, despite noise increase. Calcium structures were best imaged at 100 kVp, iodine-blood mixtures at 80 kVp. These results suggest that in small-sized objects, lower tube voltages are advantageous for visualizing high-contrast targets, due to the stronger photoelectric effect enhancing tissue attenuation differences. However, for soft tissue structures with inherently low contrast, the benefits of increased photon flux and reduced noise at higher tube voltages clearly outweigh the contrast limitations. As the used SSDE is an estimate based on the CTDI_Vol it can also be used to determine the effective dose by multiplication of the SSDE with the scan length and the age and scan region-based conversion factor. This in mind we anticipate that usage of the scenario specific best fitting kVp can result in longer usage of lower CTDI_Vols and thus yield lower effective doses for adults and children.

In the large phantom, due to higher absorption and scatter, few photons reached the detector at low kVp. Thus, higher tube voltages consistently produced superior image quality. For iodine-blood mixtures, 140 kVp gave the best CNR up to 3 mGy SSDE; above that, 120 kVp outperformed 140 kVp. This crossover effect reflects the increasing dominance of contrast loss at very high photon energies (from Compton scattering) as image noise becomes less of a limiting factor with rising dose. Iodine contrast benefits from low kVp are mainly due to the x-ray spectrum’s intensity peak being near the material specific k-edge [10, 11]. This effect becomes less relevant for calcium or soft tissue such that high kVp scanning prevails through noise suppression. Accordingly, 140 kVp provided the highest CNRs in the large phantom at low to moderate doses. The combination of greater object thickness, increased scatter, and limited beam penetration rendered low-kVp protocols ineffective in this context.

Collectively, the results from the large phantom confirm that lower tube voltage settings are not suitable for imaging in patients with greater body diameter, regardless of the target tissue’s contrast level. Instead, the balance between photon penetration and image noise reduction clearly favors higher voltage protocols, particularly for low-contrast targets. Our results align with prior theoretical work while offering practical recommendations. Earlier studies showed that low-kVp scans yield higher noise and should preferably be for small-diameter imaging [9, 12–14]. Karmazyn et al. described noise increase in the center of phantoms with increasing diameter size and suggested optimizing kVp for expected contrast gain, while keeping CNR unchanged [9]. These works relied on CTDI_Vol_32_ (this information is available to radiologists) limiting size-specific conclusions [15]. By using SSDE, we obtained tube voltage recommendations specific to large and small patient imaging depending on TOI.

Finally, comparing iterative reconstruction (iDose(0) vs. iDose(5)) revealed ~ 34% noise reduction, similar to Song et al. (37%) [16]. Although Racine et al. noted nonlinear effects on contrast, our data showed negligible influence [17–19]. CNR trends remained similar, with amplitudes shifted upward, confirming noise reduction potential while preserving contrast.

The scanner used in this study is equipped with a dual-layer, energy-integrating detector. Photon-counting CT has demonstrated considerable potential for dose reduction while maintaining comparable image quality. It is therefore plausible that similar trends would be observed with photon-counting systems, potentially at lower dose levels. Furthermore, lower kVp settings may become more favorable in this context, given their association with reduced dose metrics. Spectral imaging techniques (e.g., monoenergetic or iodine-based reconstructions) may also provide additional value for pre-interventional planning. However, as these aspects were beyond the scope of the present study and involve longer reconstruction times, they were not included in our analysis.

Our findings are fundamentally dependent on the displayed CTDI_Vol of the CT. It is known that CT scanners include an inherent variation of the displayed dose and the in reality administered dose. The specifications for constancy tests limit the relative deviation to plus or minus 20% or to an absolute value of plus minus 1 mGy. This affects our data with a certain uncertainty as the displayed dose value can differ from the real dose value, explicitly for low dose values.

### Limitations

Our research inherits certain limitations: As the main CT dose value available for interventionalists, the CTDI_Vol, reflects a calibration value measured in water equivalent phantoms, the used phantom with its water equivalent surrounding material was estimated fitting for our experimental design of this study. Consequently, the influence of real human body compositions needs to be addressed in future studies. The influence of slice thickness was not analyzed. Nagel et al. state that according to Brooks’ formula, dose must be doubled if slice thickness is halved [3]. At the same time, contrast increases more strongly with thinner slices, as contrast is inversely proportional to slice thickness, while noise rises only with the square root of slice thickness. How this trade-off affects our findings remains to be tested. Another limitation lies in the phantom material, which mainly corresponds to water density. Human tissues vary in density and may cause additional beam hardening. Phantom sizes represented normally built adults and children, but transferability to obese or cachectic patients is limited. Furthermore, contrast of our spectral rods was fixed (e.g., 4 mg/ml iodine-blood, 100 mg/ml calcium) and may differ for other tissue compositions. The present study did not consider artefacts caused by the needles used during CT-intervention. As such artefacts underlay the beam hardening effect the choice of tube voltage could directly influence the CNR. This needs to be further investigated in future studies.

## Conclusion

This study demonstrates that practical scan protocol recommendations for low-dose CT-guided interventions can be derived by considering both the type of target tissue and the patient’s effective body diameter. In particular, the choice of tube voltage significantly influences image quality, as reflected in the contrast-to-noise ratio.

For soft tissue targets, the effective object diameter had little to no impact on the optimal tube voltage. Across all tested sizes and dose levels, 140 kVp consistently provided the best CNR, and is therefore recommended in such cases.

For calcium-containing targets, the optimal tube voltage depends on object size. In smaller objects (≤ 20 cm diameter), 100 kVp provided the best results. However, in larger objects (≥ 35 cm), 140 kVp clearly outperformed all lower settings.

For iodine-enhanced targets, small objects should be scanned at 80 kVp to maximize contrast. In larger patients, 140 kVp is preferred at low dose levels (SSDE ≤ 3 mGy). Beyond this threshold, 120 kVp provides a better balance between contrast and noise.

These findings support the implementation of diameter- and tissue-adapted scan protocols in interventional CT to ensure optimal image quality at minimal radiation dose (see Table 2)

**Table 2:** Recommendation based on the findings of this study indicating which kVp should be used for certain dose levels depending on the TOI and the diameter of the object.

Tissue of Interest	Tube voltages for small diameter scans of ~ 20 cm	Tube voltages for large diameter scans ~ 35 cm	
Soft tissue/low contrast	140 kVp	140 kVp	
Calcium/ high contrast	100 kVp	140 kVp	
Iodine-blood	80 kVp	≤ 3 mGy	> 3 mGy
		140 kVp	120 kVp

## Data Availability

The phantom data will be made available on reasonable request from the Corresponding Author.
